# Time Trends in Cause-Specific Mortality in Patients with Pulmonary Embolism Aged 50 Years and Older

**DOI:** 10.1055/a-2668-5296

**Published:** 2025-08-08

**Authors:** Katarina Glise Sandblad, Kristina Svennerholm, Jacob Philipson, Maria Roupe, Aldina Pivodic, Andrea Dahl Sturedahl, Carl Johan Svensson, Sam Schulman, Mazdak Tavoly

**Affiliations:** 1 Department of Molecular and Clinical Medicine, Institute of Medicine, Sahlgrenska Academy, University of Gothenburg, Gothenburg, Sweden; 2 Department of Medicine, Geriatrics and Emergency Medicine, Region Västra Götaland, Sahlgrenska University Hospital/Östra, Gothenburg, Sweden; 3 Department of Anaesthesiology and Intensive Care, Institute of Clinical Sciences, Sahlgrenska Academy, University of Gothenburg, Gothenburg, Sweden; 4 Department of Anaesthesiology and Intensive Care, Region Västra Götaland, Sahlgrenska University Hospital, Gothenburg, Sweden; 5 Department of Medicine, Region Västra Götaland, Sahlgrenska University Hospital/Mölndal, Gothenburg, Sweden; 6 APNC Sweden, Gothenburg, Sweden; 7 Department of Medicine and Thrombosis and Atherosclerosis Research Institute, McMaster University, Hamilton, Ontario, Canada; 8 Department of Medicine, Geriatrics and Emergency Medicine, Region Västra Götaland, Sahlgrenska University Hospital/Sahlgrenska, Gothenburg, Sweden; 9 Department of Research, Østfold Hospital, Sarpsborg, Norway

**Keywords:** pulmonary embolism, mortality, trend, neoplasm

## Abstract

**Background:**

Patients with pulmonary embolism (PE) have high mortality rates. However, data on cause-specific mortality trends in this population are limited.

**Aims:**

To study time trends in cause-specific mortality among PE patients aged ≥50 years, analyzed across three time periods: 2006–2011, 2012–2017, and 2018–2023. The secondary aims included examining mortality trends in matched controls and subgroups of PE patients.

**Methods:**

This nationwide Swedish register study included patients with a first-time PE and matched controls. We assessed 30-day and 31- to 365-day cause-specific mortality and employed age- and sex-adjusted Poisson regression for the relative risk (RR) for annual mortality trends.

**Results:**

The study comprised 115,476 patients, with cancer as the leading cause of 30-day mortality, stable at 4.7% from 2006–2011 to 2018–2023 (RR 1.00; 95% confidence interval [CI]: 0.99–1.01). Mortality from fatal venous thromboembolism (VTE) decreased from 2.7 to 1.3% (RR 0.94; 95% CI: 0.93–0.95), and cardiovascular disease from 2.3 to 1.1% (RR 0.94; 95% CI: 0.93–0.94). The 31- to 365-day mortality from cancer was stable at 11.8% in 2006–2011 and 11.4% in 2018–2022 (RR 1.00; 95% CI: 0.99–1.00), while mortality due to cardiovascular disease decreased from 4.1 to 2.3% (RR 0.96; CI: 0.95–0.96), and fatal VTE from 0.8 to 0.5% (RR 0.95; 95%: 0.93–0.96). Subgroup analysis showed a decrease in cancer-related mortality among PE patients with known cancer, while it increased in those without known cancer.

**Conclusion:**

Cancer was the leading cause of death in PE patients aged ≥50 years, with stable rates over time due to contrasting trends in patients with and without known cancer. Fatal VTE comprised a minor percentage of overall mortality in recent years.

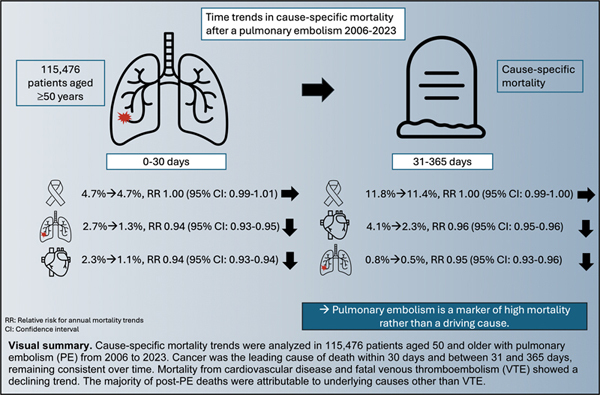

## Background

Patients suffering a first-time pulmonary embolism (PE) have high all-cause mortality rates. Although 30-day mortality following a PE has been observed to decline over time, 1-year mortality—excluding 30-day or inpatient deaths—has remained stable or shown only a slight decline. 1 2

Exploring specific mortality causes is essential for understanding these trends and identifying potential preemptive measures. Previous studies in PE patients have indicated that PE and its complications, along with cancer and infection, are the primary contributors to 30-day mortality. 3 4 In contrast, long-term mortality has been attributed to cancer, cardiovascular disease, and recurrent, fatal PE. 4 5 Additionally, bleeding and infection, 6 as well as liver failure, 5 have been reported as important contributors to death. Fatal PE showed a decline in Europe until 2015 7 and a decline in North America until 2006, after which the trends plateaued, with some demographics even experiencing an increase. 8 Beyond this, time trends for the major causes of mortality in patients with PE remain largely unknown. Considering the recent advancements in the management of acute PE, 9 the improved anticoagulant therapies with direct oral anticoagulants (DOACs), 10 11 and the declining mortality from cardiovascular diseases 12 13 alongside improvements in cancer treatment 13 14 —including novel therapies, more frequent follow-up with imaging, and prolonged survival even in advanced stages—it is reasonable to consider that the relative contributions of various causes of death in patients with PE may have shifted over time. Updated knowledge is essential for guiding the most effective future strategies to decrease mortality following a PE.

The primary aim of this study was to describe time trends in 30-day and 31- to 365-day cause-specific mortality among patients aged ≥50 years with a first-time PE diagnosis between 2006 and 2023. Secondary aims included evaluating corresponding mortality trends age-, sex-, area of residence-, and index date-matched individuals without PE, as well as in the following subgroups of PE patients: those with cancer, those without cancer, and those with temporary provoking factors. Additionally, we assessed trends in all-cause mortality across all study groups and compared mortality risks between PE patients and their matched controls.

## Methods

### Study Population

This nationwide Swedish register study included patients with a first-time PE from 2006 to 2023. Patients were identified in the National Patient Register, which was linked to the National Prescribed Drug Register and the National Cause of Death Register.

Sweden has a universal health care system providing low-cost health care to all citizens. The National Patient Register has virtually complete coverage for inpatient and outpatient hospital-based health care, but does not include primary care. 15 16 The National Prescribed Drug Register includes all prescriptions dispensed at Swedish pharmacies since July 2005. 17 The National Cause of Death Register includes underlying and contributing causes of death for all Swedish inhabitants. 18 The Total Population Register contains data on all inhabitants in Sweden, including their area of residence. 19

Data from the included registers were linked by the National Board of Health and Welfare using the unique Personal Identification Number assigned to all Swedish residents. Patients with a first-time PE, along with their comorbidities, were identified through the National Patient Register. For each case, up to five control persons were retrieved from the Total Population Register and matched by age, sex, area of residence, and the date of the case's first PE diagnosis. Control persons who developed PE during follow-up were censored at the time of diagnosis and subsequently included in the PE cohort. Data on dispensed pharmacological treatment was retrieved from the National Prescribed Drug Register, and mortality data from the National Cause of Death Register. The data was merged, and the Personal Identification Number was exchanged for a serial number to create a pseudonymized dataset.

The study included all patients with a first-time diagnosis of PE, identified by the International Classification of Diseases, 10th Revision (ICD-10) codes I26.0 and I26.9, recorded as either a primary or secondary diagnosis in outpatient or inpatient care from 2006 to 2023. For the 30-day mortality analysis, patients were included until November 30, 2023; for the 31- to 365-day analysis, patients were included until December 31, 2022. For analysis of the total number of deaths and cause-specific deaths within 365 days of a PE, patients were included until December 31, 2023.

### Exclusion Criteria

For PE patients:

1. Prescription of anticoagulant treatment dispensed within 6 months prior to venous thromboembolism (VTE).
2. Patients younger than 50 years at the time of first VTE diagnosis.
3. Missing demographics data or data inconsistencies considering dates.

For PE controls:

1. Anticoagulant medication dispensed within 6 months of corresponding PE patient index date.

**Fig. 1:**
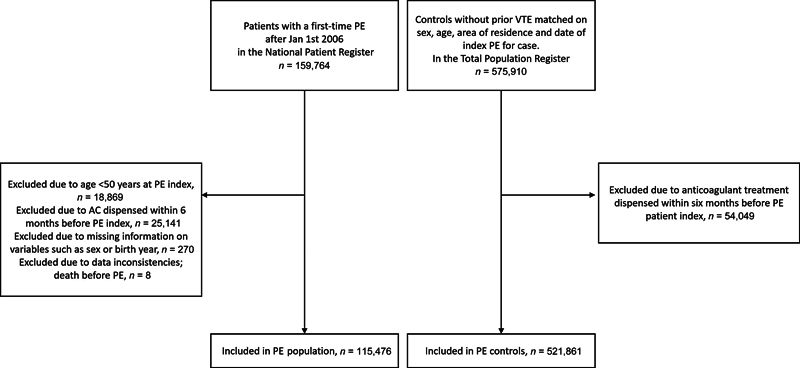
Flow chart for study inclusion of patients with pulmonary embolism and matched controls. PE, Pulmonary embolism. AC, anticoagulant medication.

See Fig. 1 for the flowchart for study inclusion.

The study was approved by the Swedish Ethical Review Authority (Dnr 2019–01956).

### Comorbidities

Comorbidities were included if registered in the National Patient Register within 7 years or on the same date as the PE for patients and on the date of the PE of the corresponding case for controls. The following comorbidities were registered: Cancer, ischemic heart disease, heart failure, peripheral artery disease, ischemic stroke, hemorrhagic stroke, dementia, chronic obstructive pulmonary disease (COPD), systemic connective tissue disorders, peptic ulcer, liver disease, diabetes mellitus, kidney failure, HIV infection, psychosis, and previous bleeding. The following temporary provoking factors were included if registered within 3 months prior to the PE date: surgery, lower extremity fracture, trauma, and oral contraceptives. For patients and controls included in 2020 or after, Covid-19 was included as a temporary factor. ICD-10 codes for all comorbidities and temporary provoking factors are provided in Supplementary Table S1 (available in the online version). Concomitant medications included antiplatelet treatment, proton pump inhibitors (PPI), statins, and selective serotonin reuptake inhibitors (SSRI) dispensed within 3 months after the PE diagnosis. For ATC codes, see Supplementary Table S1 (available in the online version).

**Table 1:** Baseline characteristics for patients with pulmonary embolism (PE) during three different time periods: 2006–2011, 2012–2017, and 2018–2023

	2006–2011	2012–2017	2018–2023
Number of PE cases	*N* = 31,050	*N* = 38,744	*N* = 45,682
Age, median (range)	75 (50–104)	74 (50–105)	74 (50–109)
**Age category**			
50–64	6,972 (22.5%)	8,259 (21.3%)	10,147 (22.2%)
65–79	13,287 (42.8%)	18,105 (46.7%)	21,692 (47.5%)
≥80	10,791 (34.8%)	12,380 (32.0%)	13,843 (30.3%)
Sex			
Female	16,269 (52.4%)	19,833 (51.2%)	23,018 (50.4%)
**Comorbidities**			
Cancer	10,013 (32.2%)	12,853 (33.2%)	13,944 (30.5%)
Ischemic heart disease	6,212 (20.0%)	5,961 (15.4%)	4,163 (9.1%)
Heart failure	5,148 (16.6%)	4,636 (12.0%)	2,757 (6.0%)
Peripheral arterial disease	912 (2.9%)	1,068 (2.8%)	906 (2.0%)
Ischemic stroke	2,553 (8.2%)	2,172 (5.6%)	1,368 (3.0%)
Hemorrhagic stroke	589 (1.9%)	764 (2.0%)	605 (1.3%)
Dementia	1,339 (4.3%)	1,535 (4.0%)	1,207 (2.6%)
COPD	3,338 (10.8%)	3,882 (10.0%)	3,377 (7.4%)
Systemic connective tissue disorders	1,436 (4.6%)	1,626 (4.2%)	1,438 (3.1%)
Peptic ulcer	390 (1.3%)	323 (0.8%)	126 (0.3%)
Liver disease	398 (1.3%)	495 (1.3%)	497 (1.1%)
Diabetes	3,923 (12.6%)	4,988 (12.9%)	4,632 (10.1%)
Renal disease	1,538 (5.0%)	2,222 (5.7%)	2,046 (4.5%)
HIV	20 (0.1%)	24 (0.1%)	57 (0.1%)
Bleeding	1,529 (4.9%)	1,569 (4.0%)	1,400 (3.1%)
Psychosis	88 (0.3%)	86 (0.2%)	109 (0.2%)
**Temporary provoking factors**			
Any temporary provoking factor	9,181 (29.6%)	11,181 (28.9%)	14,616 (32.0%)
Covid			2,343 (5.1%)
Surgery	6,923 (22.3%)	8,584 (22.2%)	9,993 (21.9%)
Lower extremity fracture	1,220 (3.9%)	1,071 (2.8%)	1,115 (2.4%)
Trauma	1,446 (4.7%)	1,781 (4.6%)	2,076 (4.5%)
Hormone replacement therapy	1,649 (5.3%)	1,888 (4.9%)	2,080 (4.6%)
Anticoagulant treatment*			
Warfarin	16,041 (51.7%)	10,532 (27.2%)	683 (1.5%)
Apixaban	0 (0.0%)	4,429 (11.4%)	18,318 (40.1%)
Rivaroxaban	0 (0.0%)	5,740 (14.8%)	7,565 (16.6%)
Edoxaban	0 (0.0%)	6 (0.0%)	566 (1.2%)
Dabigatran	1 (0.0%)	245 (0.6%)	490 (1.1%)
Low-molecular-weight heparin	15,502 (49.9%)	16,853 (43.5%)	11,795 (25.8%)
**Other concomitant treatment**			
Antiplatelet treatment	3,373 (10.9%)	3,618 (9.3%)	3,457 (7.6%)
Proton pump inhibitor	7,722 (24.9%)	11,343 (29.3%)	15,214 (33.3%)
Statins	4,483 (14.4%)	6,285 (16.2%)	9,055 (19.8%)
Selective serotonin reuptake inhibitors	3,172 (10.2%)	3,820 (9.9%)	4,032 (8.8%)

Abbreviation: PE, pulmonary embolism.

Note: Data are presented as median (range) and number of observations, or number (percentage).

* Dispensed within 30 days from PE diagnosis.

### Outcomes

The primary outcome of this study was cause-specific 30-day and 31- to 365-day mortality, defined as the primary (underlying) cause of death. The secondary outcome was all-cause mortality.

Initially, the 20 most common mortality causes for the entire group were identified to ensure no important causes were overlooked. The mortality causes were then grouped according to the World Health Organization's list of mortality causes and corresponding ICD-10 codes, 20 including neuropsychiatric conditions (dementia, psychiatric illness, and neurological illness), cardiovascular diseases, respiratory diseases, malignant neoplasms (here referred to as cancer), injuries, infectious and parasitic diseases, and Covid-19 with the addition of two extra groups: fatal VTE and bleeding. ICD-10 codes for mortality causes are presented in Supplementary Table S2 (available in the online version).

**Table 2:** 30-day and 31 to 365-day mortality causes in different time periods. Data presented with number of events, percentage, and event rate per 1000 person-years

Mortality cause		2006–2011	2012–2017	2018–2023
Cancer	30-day mortality			
	*n* / *N* (%)	1,452/31,050	1,862/38,744	2,147/45,652
		(4.7%)	(4.8%)	(4.7%)
	Event rate (95% CI) per 1,000 person-years	600.9 (570.4–632.6)	606.0 (578.8–634.1)	590.5 (565.7–616.0)
	31- to 365-day mortality				
	*n* / *N* (%)	3,188/26,918	4,281/34,400	3,981/34,834
		(11.8%)	(12.4%)	(11.4%)
	Event rate (95% CI)***	150.2 (145.0–155.5)	157.8 (153.1–162.6)	143.3 (138.9–147.8)
Cardiovascular diseases	30-day mortality			
	*n* / *N* (%)	722/31,050	591/38,744	490/45,652
		(2.3%)	(1.5%)	(1.1%)
	Event rate (95% CI)***	298.8 (277.4–321.4)	192.3 (177.1–208.5)	134.8 (123.1–147.2)
	31- to 365-day mortality				
	*n* / *N* (%)	1,114/26,918	1,099/34,400	813/34,834
		(4.1%)	(3.2%)	(2.3%)
	Event rate (95% CI)***	52.5 (49.5–55.7)	40.5 (38.1–43.0)	29.3 (27.3–31.3)
Respiratory diseases	30-day mortality			
	*n* / *N* (%)	139/31,050	213/38,744	307/45,652
		(0.4%)	(0.5%)	(0.7%)
	Event rate (95% CI)***	57.5 (48.4–67.9)	69.3 (60.3–79.3)	84.4 (75.2–94.4)
	31- to 365-day mortality				
	*n* / *N* (%)	300/26,918	425/34,400	393/34,834
		(1.1%)	(1.2%)	(1.1%)
	Event rate (95% CI)***	14.1 (12.6–15.8)	15.7 (14.2–17.2)	14.1 (12.8–15.6)
Neuropsychiatric conditions**	30-day mortality			
	*n* / *N* (%)	113/31,050	149/38,744	214/45,652
		(0.4%)	(0.4%)	(0.5%)
	Event rate (95% CI)***	46.8 (38.5–56.2)	48.5 (41.0–56.9)	58.9 (51.2–67.3)
	31- to 365-day mortality				
	*n* / *N* (%)	158/26,918	307/34,400	373/34,834
		(0.6%)	(0.9%)	(1.1%)
	Event rate (95% CI)***	7.4 (6.3–8.7)	11.3 (10.1–12.7)	13.4 (12.1–14.9)
Fatal venous thromboembolism	30-day mortality			
	*n* / *N* (%)	825/31,050	723/38,744	600/45,652
		(2.7%)	(1.9%)	(1.3%)
	Event rate (95% CI)***	341.4 (318.5–365.5)	235.3 (218.5–253.1)	165.0 (152.1–178.8)
	31–365-day mortality				
	*n* / *N* (%)	219/26,918	227/34,400	157/34,834
		(0.8%)	(0.7%)	(0.5%)
	Event rate (95% CI)***	10.3 (9.0–11.8)	8.4 (7.3–9.5)	5.7 (4.8–6.6)
Bleeding	30-day mortality			
	*n* / *N* (%)	50/31,050	53/38,744	72/45,652
		(0.2%)	(0.1%)	(0.2%)
	Event rate (95% CI)***	20.7 (15.4–27.3)	17.2 (12.9–22.6)	19.8 (15.5–24.9)
	31- to 365-day mortality				
	*n* / *N* (%)	56/26,918	73/34,400	79/34,834
		(0.2%)	(0.2%)	(0.2%)
	Event rate (95% CI)***	2.6 (2.0–3.4)	2.7 (2.1–3.4)	2.8 (2.3–3.5)
Injury	30-day mortality			
	*n* / *N* (%)	276/31,050	207/38,744	193/45,652
		(0.9%)	(0.5%)	(0.4%)
	Event rate (95% CI)***	114.2 (101.1–128.5)	67.4 (58.5–77.2)	53.1 (45.9–61.1)
	31- to 365-day mortality				
	*n* / *N* (%)	96/26,918	101/34,400	111/34,834
		(0.4%)	(0.3%)	(0.3%)
	Event rate (95% CI)***	4.5 (3.7–5.5)	3.7 (3.0–4.5)	4.0 (3.3–4.8)
Infectious and parasitic diseases	30-day mortality			
	*n* / *N* (%)	84/31,050	106/38,744	95/45,652
		(0.3%)	(0.3%)	(0.2%)
	Event rate (95% CI)***	34.8 (27.7–43.0)	34.5 (28.2–41.7)	26.1 (21.1–31.9)
	31- to 365-day mortality				
	*n* / *N* (%)	102/26,918	118/34,400	102/34,834
		(0.4%)	(0.3%)	(0.3%)
	Event rate (95% CI)***	4.8 (3.9–5.8)	4.3 (3.6–5.2)	3.7 (3.0–4.5)
Covid-19	30-day mortality			
	*n* / *N* (%)	0	0	392/45,652
				(0.9%)
	Event rate (95% CI)***	0	0	107.8 (97.4–119.0)
	31–365-day mortality				
	*n* / *N* (%)	0	0	200/34,834
				(0.6%)
	Event rate (95% CI)***	0	0	7.2 (6.2–8.3)
Other causes	30-day mortality			
	*n* / *N* (%)	471/31,050	440/38,744	524/45,652
		(1.5%)	(1.1%)	(1.1%)
	Event rate (95% CI)***	194.9 (177.7–213.3)	143.2 (130.1–157.2)	144.1 (132.0–157.0)
	31- to 365-day mortality				
	*n* / *N* (%)	509/26,918	627/34,400	540/34,834
		(1.9%)	(1.8%)	(1.6%)
	Event rate (95% CI)***	24.0 (21.9–26.2)	23.1 (21.3–25.0)	19.4 (17.8–21.1)
All-cause mortality	30-day mortality			
	*n* / *N* (%)	4,132/31,050	4,344/38,744	5,034/45,652
		(13.3%)	(11.2%)	(11.0%)
	Event rate (95% CI)***	1,710.0 (1,658.3–1,763.0)	1,413.7 (1,372.0–1,456.4)	1,384.4 (1,346.4–1,423.2)
	31–365-day mortality				
	*n* / *N* (%)	5,742/26,918	7,258/34,400	6,749/34,834
		(21.3%)	(21.1%)	(19.4%)
	Event rate (95% CI)***	270.6 (263.6–277.6)	267.5 (261.4–273.7)	242.9 (237.2–248.8)

Notes: Confidence interval for unadjusted event rates per 1,000 person-years are obtained from exact Poisson confidence limits.

*For 31- to 365-day mortality, only numbers for 2008–2022, not 2023. **Dementia, psychiatric illness, neurological illness. *** Per 1,000 person-years.

### Statistical Analysis

Baseline characteristics are described with median and range for continuous variables and frequency and percentage for categorical variables. Patients were categorized into three groups based on the date of their first PE: 2006–2011, 2012–2017, and 2018–2023.

Mortality rates were calculated as the number of deaths divided by the number of follow-up years for each study group and expressed in 1,000 person-years. The 95% confidence interval (CI) was calculated using exact Poisson limits. Cause-specific mortality rates for 30 days and 31 to 365 days were analyzed across the different time periods. Poisson regression was used to determine the relative risk (RR) for temporal changes in 30-day and 31- to 365-day mortality, adjusted for age and sex. Separate analyses were conducted for matched controls, PE patients with cancer, PE patients without cancer, and PE patients with temporary provoking factors. Temporary provoking factors included Covid-19, recent surgery, lower extremity fracture, trauma, and hormone replacement therapy.

Cox regression was used to estimate the relative risk of mortality for cases versus controls. The proportional hazards assumption was checked and confirmed. In the first model, analyses were adjusted for age and sex. The second model included additional clinically relevant confounders and predictors. Selection of adjustment variables was based on the number of events, applying the rule of one variable per 10 events, and their strength of association in the unadjusted analyses. Results are presented as hazard ratios (HRs) with corresponding 95% CIs.

Patients were followed until death or up to 30 days or 1 year after VTE, respectively, whichever came first.

All tests were two-tailed. Due to the multiple analyses performed and the large number of patients included in this study, a significance level of 0.0001 was applied. All analyses were performed using SAS software version 9.4 (SAS Institute Inc., Cary, North Carolina, United States).

## Results

Between 2006 and 2023, 115,476 patients ≥50 years of age with first-time PE were included. The median age was 74 years (interquartile range: 66–82), and 51% were female.

Baseline characteristics among PE patients changed over the study period, with a decreasing prevalence of most comorbidities, including ischemic heart disease (from 20.0% in 2006–2011 to 9.1% in 2018–2023), heart failure (from 16.6 to 6.0%), ischemic stroke, COPD, dementia, diabetes, and previous bleeding ( Table 1 ). Cancer remained the most common comorbidity, with a stable prevalence of 32.2 to 30.5% over time. Among temporary provoking factors, no major changes were observed except for Covid-19, which did not exist before the last time period.

Baseline characteristics among PE controls also changed over the study period ( Supplementary Table S3 , available in the online version). Ischemic heart disease decreased from 11.1% in 2006–2011 to 6.6% in 2018–2023, and heart failure from 4.6 to 2.0%. In contrast, the prevalence of cancer remained stable, ranging from 12.4 to 14.7%.

**Table 3:** Hazard ratios for cause-specific and all-cause 30-day and 31 to 365-day mortality for PE patients versus controls. Age-and sex adjusted estimated as well as multivariable adjusted hazard ratios

Mortality cause		2006–2011	2012–2017	2018–2023
Cancer	30-day mortality			
	*n* / *N* (%)	1,452/31,050	1,862/38,744	2,147/45,652
		(4.7%)	(4.8%)	(4.7%)
	Event rate (95% CI) per 1,000 person-years	600.9 (570.4–632.6)	606.0 (578.8–634.1)	590.5 (565.7–616.0)
	31- to 365-day mortality				
	*n* / *N* (%)	3,188/26,918	4,281/34,400	3,981/34,834
		(11.8%)	(12.4%)	(11.4%)
	Event rate (95% CI)***	150.2 (145.0–155.5)	157.8 (153.1–162.6)	143.3 (138.9–147.8)
Cardiovascular diseases	30-day mortality			
	*n* / *N* (%)	722/31,050	591/38,744	490/45,652
		(2.3%)	(1.5%)	(1.1%)
	Event rate (95% CI)***	298.8 (277.4–321.4)	192.3 (177.1–208.5)	134.8 (123.1–147.2)
	31- to 365-day mortality				
	*n* / *N* (%)	1,114/26,918	1,099/34,400	813/34,834
		(4.1%)	(3.2%)	(2.3%)
	Event rate (95% CI)***	52.5 (49.5–55.7)	40.5 (38.1–43.0)	29.3 (27.3–31.3)
Respiratory diseases	30-day mortality			
	*n* / *N* (%)	139/31,050	213/38,744	307/45,652
		(0.4%)	(0.5%)	(0.7%)
	Event rate (95% CI)***	57.5 (48.4–67.9)	69.3 (60.3–79.3)	84.4 (75.2–94.4)
	31- to 365-day mortality				
	*n* / *N* (%)	300/26,918	425/34,400	393/34,834
		(1.1%)	(1.2%)	(1.1%)
	Event rate (95% CI)***	14.1 (12.6–15.8)	15.7 (14.2–17.2)	14.1 (12.8–15.6)
Neuropsychiatric conditions**	30-day mortality			
	*n* / *N* (%)	113/31,050	149/38,744	214/45,652
		(0.4%)	(0.4%)	(0.5%)
	Event rate (95% CI)***	46.8 (38.5–56.2)	48.5 (41.0–56.9)	58.9 (51.2–67.3)
	31- to 365-day mortality				
	*n* / *N* (%)	158/26,918	307/34,400	373/34,834
		(0.6%)	(0.9%)	(1.1%)
	Event rate (95% CI)***	7.4 (6.3–8.7)	11.3 (10.1–12.7)	13.4 (12.1–14.9)
Fatal venous thromboembolism	30-day mortality			
	*n* / *N* (%)	825/31,050	723/38,744	600/45,652
		(2.7%)	(1.9%)	(1.3%)
	Event rate (95% CI)***	341.4 (318.5–365.5)	235.3 (218.5–253.1)	165.0 (152.1–178.8)
	31- to 365-day mortality				
	*n* / *N* (%)	219/26,918	227/34,400	157/34,834
		(0.8%)	(0.7%)	(0.5%)
	Event rate (95% CI)***	10.3 (9.0–11.8)	8.4 (7.3–9.5)	5.7 (4.8–6.6)
Bleeding	30-day mortality			
	*n* / *N* (%)	50/31,050	53/38,744	72/45,652
		(0.2%)	(0.1%)	(0.2%)
	Event rate (95% CI)***	20.7 (15.4–27.3)	17.2 (12.9–22.6)	19.8 (15.5–24.9)
	31- to 365-day mortality				
	*n* / *N* (%)	56/26,918	73/34,400	79/34,834
		(0.2%)	(0.2%)	(0.2%)
	Event rate (95% CI)***	2.6 (2.0–3.4)	2.7 (2.1–3.4)	2.8 (2.3–3.5)
Injury	30-day mortality			
	*n* / *N* (%)	276/31,050	207/38,744	193/45,652
		(0.9%)	(0.5%)	(0.4%)
	Event rate (95% CI)***	114.2 (101.1–128.5)	67.4 (58.5–77.2)	53.1 (45.9–61.1)
	31- to 365-day mortality				
	*n* / *N* (%)	96/26,918	101/34,400	111/34,834
		(0.4%)	(0.3%)	(0.3%)
	Event rate (95% CI)***	4.5 (3.7–5.5)	3.7 (3.0–4.5)	4.0 (3.3–4.8)
Infectious and parasitic diseases	30-day mortality			
	*n* / *N* (%)	84/31,050	106/38,744	95/45,652
		(0.3%)	(0.3%)	(0.2%)
	Event rate (95% CI)***	34.8 (27.7–43.0)	34.5 (28.2–41.7)	26.1 (21.1–31.9)
	31- to 365-day mortality				
	*n* / *N* (%)	102/26,918	118/34,400	102/34,834
		(0.4%)	(0.3%)	(0.3%)
	Event rate (95% CI)***	4.8 (3.9–5.8)	4.3 (3.6–5.2)	3.7 (3.0–4.5)
Covid-19	30-day mortality			
	*n* / *N* (%)	0	0	392/45,652
				(0.9%)
	Event rate (95% CI)***	0	0	107.8 (97.4–119.0)
	31- to 365-day mortality				
	*n* / *N* (%)	0	0	200/34,834
				(0.6%)
	Event rate (95% CI)***	0	0	7.2 (6.2–8.3)
Other causes	30-day mortality			
	*n* / *N* (%)	471/31,050	440/38,744	524/45,652
		(1.5%)	(1.1%)	(1.1%)
	Event rate (95% CI)***	194.9 (177.7–213.3)	143.2 (130.1–157.2)	144.1 (132.0–157.0)
	31- to 365-day mortality				
	*n* / *N* (%)	509/26,918	627/34,400	540/34,834
		(1.9%)	(1.8%)	(1.6%)
	Event rate (95% CI)***	24.0 (21.9–26.2)	23.1 (21.3–25.0)	19.4 (17.8–21.1)
All-cause mortality	30-day mortality			
	*n* / *N* (%)	4,132/31,050	4,344/38,744	5,034/45,652
		(13.3%)	(11.2%)	(11.0%)
	Event rate (95% CI)***	1,710.0 (1,658.3–1,763.0)	1,413.7 (1,372.0–1,456.4)	1,384.4 (1,346.4–1,423.2)
	31- to 365-day mortality				
	*n* / *N* (%)	5,742/26,918	7,258/34,400	6,749/34,834
		(21.3%)	(21.1%)	(19.4%)
	Event rate (95% CI)***	270.6 (263.6–277.6)	267.5 (261.4–273.7)	242.9 (237.2–248.8)

Notes: Confidence interval for unadjusted event rates per 1,000 person-years are obtained from exact Poisson confidence limits.

*For 31- to 365-day mortality, only numbers for 2008–2022, not 2023. **Dementia, psychiatric illness, neurological illness. *** Per 1,000 person-years.

As expected, anticoagulant treatment patterns among PE patients changed over time, with decreasing use of warfarin and low-molecular-weight heparin and increasing use of DOACs, in particular apixaban. Among concomitant treatments, statin and proton pump inhibitor use increased, while antiplatelet treatment declined over time.

**Fig. 2:**
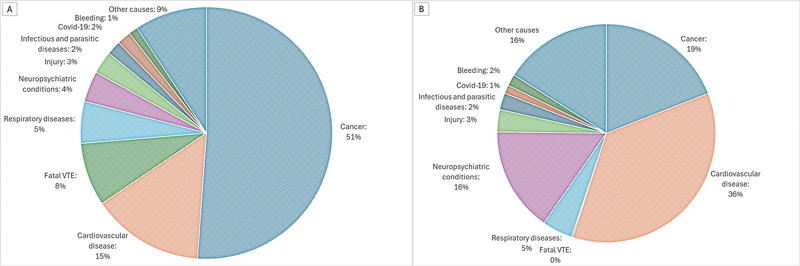
Mortality groups, deaths within 365 days for the entire study period, 2006-2023, for PE patients ( **A** ) and PE controls ( **B** ). VTE, venous thromboembolism.

Among all individuals included in the study through December 31, 2023, a total of 34,072 PE patients (29.5%) and 16,251 controls (3.1%) died within 365 days of the index date. The primary causes of death among PE patients were cancer (17,428 deaths; 51% of all deaths), cardiovascular disease (4,911 deaths; 15%), and fatal VTE (2,769 deaths; 8%) ( Fig. 2A ). For other causes of death, see Fig. 2A . The most common causes of death were cardiovascular disease (5,842 deaths; 36%), cancer (3,097 deaths; 19%), and neuropsychiatric—including both neurological and psychiatric disorders—accounting for 2,523 deaths (16%) ( Fig. 2B ). Among all individuals included up to December 31, 2022, and thereby at risk of death during both the 0- to 30-day and 31- to 365-day intervals, 32,540 PE patients and 15,795 controls died. Of these, 39% of PE patients and 9% of controls died within 30 days following the index date, whereas 61% of PE patients and 91% of controls died within 31 to 365 days.

### 30-day Mortality

**Fig. 3:**
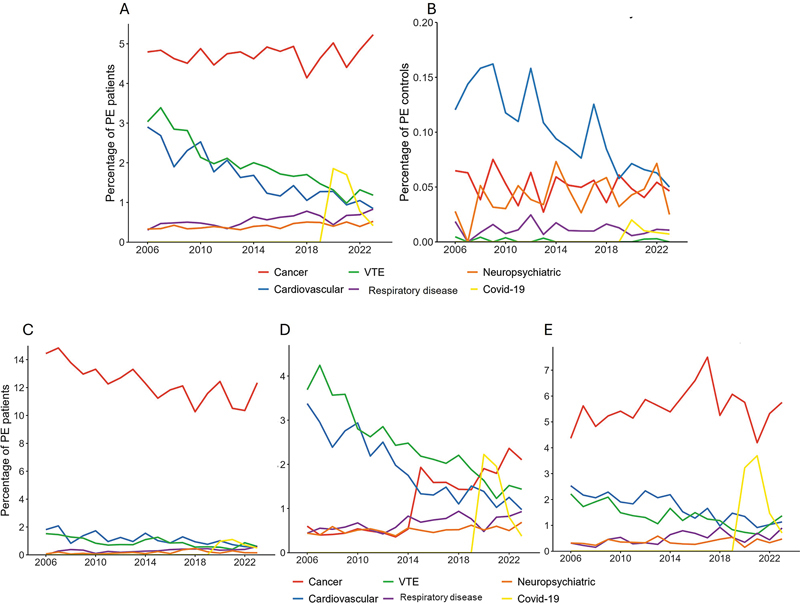
Percentage of patients 50 years and older with first-time pulmonary embolism (PE) who died within 0-30 days with the leading causes of death – cancer, cardiovascular disease, venous thromboembolism (VTE), chronic lung disease, neuropsychiatric diseases (including dementia, neurologic diseases, and psychiatric disorders), and Covid-19. **A** . PE patients. **B** . Matched controls. **C** . PE patients with cancer. **D** . PE patients without cancer. **E** . PE patients with temporary provoking factors.

The leading cause of 30-day mortality was cancer, which remained stable at 4.7% in 2006–2011 and 4.7% of all PE patients in 2018–2023 (RR 1.00; 95% CI: 0.99–1.01; *p* = 0.96) ( Table 2 , Fig. 3A , Supplementary Table S4 , available in the online version). Fatal VTE decreased from 2.7 to 1.3% (RR 0.94; 95% CI: 0.93–0.95; *p* < 0.0001), and cardiovascular mortality from 2.3 to 1.1% (RR 0.94; 95% CI: 0.93–0.94; *p* < 0.0001).

Mortality from respiratory diseases increased from 0.4 to 0.7% (RR 1.04; 95% CI: 1.02–1.05; *p* < 0.0001), while neuropsychiatric death was stable at 0.4 to 0.5% (RR 1.02; 95% CI: 1.00–1.04; *p* = 0.017). Mortality from injury was low and decreasing over the study period from 0.9 to 0.4% (RR 0.94; 95% CI: 0.92–0.95; *p* < 0.0001). Mortality from bleeding was low throughout the study period at 0.2% (RR 0.99; 95% CI: 0.97–1.02; *p* = 0.67). Covid-19 contributed to mortality only in 2018 to 2023 (0.9%).

Overall, the 30-day (all-cause) mortality decreased over time, from 13.3% in 2006–2011 to 11.0% in 2018–2023 (RR 0.98; 95% CI: 0.98–0.99; *p* < 0.0001).

Among controls, the leading cause of death was cardiovascular disease, declining from 0.1% in 2006–2011 to 0.07% in 2018–2023 (RR 0.95; 95% CI: 0.94–0.97; *p* < 0.0001) ( Fig. 3B , Supplementary Tables S4 and S5 , available in the online version). Cancer was the second leading cause of death and remained stable over time. All-cause mortality also remained relatively stable, decreasing from 0.3% in 2006–2011 to 0.2% in 2018–2023.

Among PE patients with cancer, the predominant cause of death was cancer, which declined from 13.5% in 2006–2011 to 11.2% in 2018–2023 (RR 0.98; 95% CI: 0.98–0.99; *p* < 0.0001) ( Fig. 3C , Supplementary Tables S4 and S6 , available in the online version). All-cause mortality in this group declined from 18.4 to 15.3% over the same time period.

Among PE patients without cancer, the leading cause of death at the beginning of the study period was fatal VTE, which declined from 3.4% in 2006–2011 to 1.6% in 2018–2023 (RR 0.94; 95% CI: 0.93–0.95; *p* < 0.0001) ( Fig. 3D , Supplementary Tables S4 and S7 , available in the online version). Cardiovascular mortality also declined during this period. In contrast, cancer-related mortality increased from 0.5 to 1.8% (RR 1.12; 95% CI: 1.10–1.13; *p* < 0.0001), becoming the leading cause of death by the end of the study period. All-cause mortality declined from 10.9 to 9.2% between 2006–2011 and 2018–2023.

Among PE patients with temporary provoking factors, cancer was the leading cause of death and remained stable over time, with mortality rates of 5.1% in 2006–2011 and 5.3% in 2018–2023 ( Fig. 3E , Supplementary Tables S4 and S8 , available in the online version). All-cause mortality declined in this group from 14.6 to 13.0% over the same period.

For 30-day cause-specific mortality estimates per time period across all PE patients, matched controls, and subgroups, see Supplementary Fig. S1 (available in the online version).

The aHR for all-cause 30-day mortality in PE patients compared with controls was 37.7 (95% CI 34.1–41.7) in 2006–2011 and increased to 41.7 (95% CI: 37.9–45.8) in 2018–2023 ( Table 3 ). For cancer-related 30-day mortality, the aHR increased from 41.6 (95% CI: 33.1–52.3) to 71.3 (95% CI: 58.0–87.6) over the study period. In contrast, cardiovascular mortality remained stable, with an aHR consistently around 14.

### 31- to 365-day Mortality

**Fig. 4:**
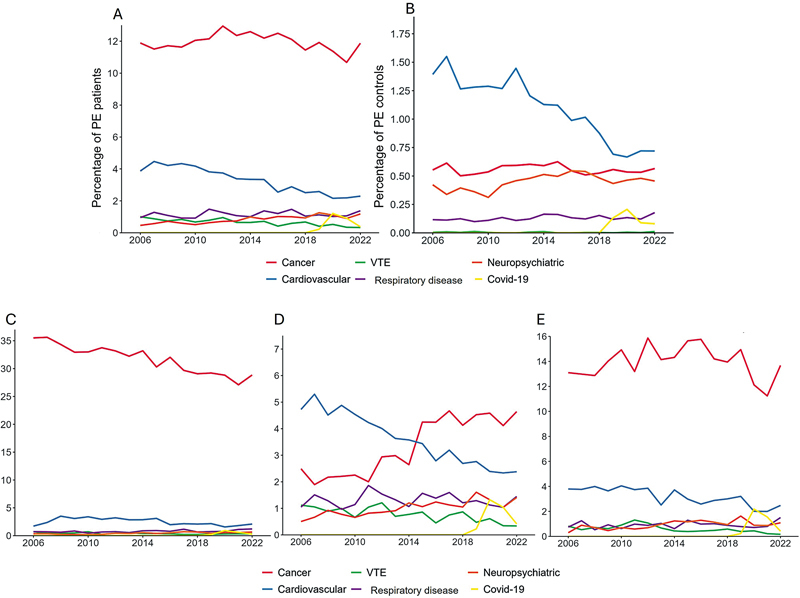
Percentage of patients 50 years and older with first-time pulmonary embolism (PE) who died within 31-365 days with the leading causes of death – cancer, cardiovascular disease, venous thromboembolism (VTE), chronic lung disease, neuropsychiatric diseases (including dementia, neurologic diseases, and psychiatric disorders), and Covid-19. **A** . PE patients. **B** . Matched controls. **C** . PE patients with cancer. **D** . PE patients without cancer. **E** . PE patients with temporary provoking factors.

The leading cause of 31- to 365-day mortality was cancer, at 11.8% in 2006–2011 and 11.4% of all PE patients in 2018 to 2022 (RR for yearly change 1.00; 95% CI: 0.99–1.00; *p* = 0.037), while mortality due to cardiovascular disease decreased from 4.1 to 2.3% (RR 0.96; 95% CI: 0.95–0.96; *p* < 0.0001) ( Table 2 , Fig. 4 , Supplementary Table S4 , available in the online version). Death from respiratory diseases was stable at 1.1% (RR 1.01; 95% CI: 0.99–1.02; *p* = 0.34), while fatal VTE decreased from 0.8 to 0.3% (RR 0.95; 95% CI: 0.93–0.96; *p* < 0.0001). Neuropsychiatric death increased from 0.6 to 1.1% (RR 1.05; 95% CI: 1.04–1.07; *p* < 0.0001). Mortality from injury was stable at 0.4 to 0.3% (RR 0.99; 95% CI: 0.97–1.01; *p* = 0.32), as well as mortality from bleeding at 0.2% (RR 1.00; 95% CI: 0.97–1.02; *p* = 0.78).

Overall, the 31- to 365-day (all-cause) mortality decreased from 21.3% in 2006–2011 to 19.4% in 2018–2022 (RR 0.99; 95% CI: 0.99–0.99; *p* < 0.0001).

Among controls, the leading cause of death was cardiovascular disease, which declined from 1.3% in 2006–2011 to 0.7% in 2018–2022 (RR 0.95; 95% CI: 0.94–0.97; *p* < 0.0001) ( Fig. 4B , Supplementary Tables S4 and S5 , available in the online version). Cancer was the second leading cause of death and showed no substantial change over time. All-cause mortality exhibited a modest decline, from 3.1% in 2006–2011 to 2.6% in 2018–2023.

Among PE patients with cancer, the most common cause of death was cancer, which declined from 34.1% in 2006–2011 to 28.6% in 2018–2023 (RR 0.98; 95% CI: 0.98–0.99; *p* < 0.0001) ( Fig. 4C , Supplementary Tables S4 and S6 , available in the online version). All-cause mortality in this group decreased from 41.0 to 34.9% over the same period.

Among PE patients without cancer, cardiovascular disease was the leading cause of death at the beginning of the study period, decreasing from 4.7% in 2006–2011 to 2.5% in 2018–2023 (RR 0.96; 95% CI: 0.95–0.96; *p* < 0.0001) ( Fig. 4D , Supplementary Tables S4 and S7 , available in the online version). In contrast, cancer-related mortality increased from 2.2% in 2006–2011 to 4.4% in 2018–2023 (RR 1.06; 95% CI: 1.05–1.07; *p* < 0.0001), becoming the leading cause of death by the end of the study period. All-cause mortality did not vary significantly, remaining at 12.8 and 13.0%.

Among PE patients with temporary provoking factors, cancer remained the leading cause of death, and was essentially unchanged over time at 13.6% in 2006–2011 and 13.0% in 2018–2023 ( Fig. 4E , Supplementary Tables S4 and S8 , available in the online version). Similarly, all-cause mortality showed minimal variation, declining slightly from 22.8 to 21.8%. The 31- to 365-day mortality estimates for different time periods for all PE patients, controls, and subgroups are presented in Supplementary Fig. S2 (available in the online version).

The aHR for all-cause 31- to 365-day mortality for PE patients compared with controls was 5.9 (95% CI: 5.7–6.2) in 2006–2011 and 6.9 (95% CI: 6.6–7.2) in 2018–2023 ( Table 3 ). For cancer-related mortality, the 31- to 365-day mortality increased from 13.9 (95% CI: 12.8–15.0) to 16.8 (95% CI: 15.6–18.1) over the study period. In contrast, cardiovascular mortality remained stable, with an aHR consistently around 3.

## Discussion

This study explored trends in cause-specific mortality in first-time PE patients ≥50 years of age over a 15-year period. Cancer was observed as the primary cause of death for 30-day and 31- to 365-day mortality, with a stable rate over time. However, subgroup analyses revealed that this apparent stability was driven by divergent trends: a decline in cancer-related mortality among PE patients with known cancer at baseline, and a concomitant increase in mortality among those without baseline cancer. Fatal VTE was a significant cause of 30-day mortality but decreased by 50% over the study period. A similar trend was observed for mortality caused by cardiovascular diseases, mirroring the reduction observed in matched controls. Death due to cardiovascular disease and fatal VTE also decreased over time when analyzing the 31- to 365-day period following PE, although in this time frame, fatal VTE was no longer a major contributor to mortality. Comparing mortality among PE patients and controls, the relative all-cause mortality risk was approximately 35 times higher in PE patients during the 30-day follow-up and approximately 6 times higher during the 31- to 365-day follow-up.

Numerous studies have established the role of cancer as a key mortality driver in VTE patients. 3 4 5 6 21 The prevalence of cancer in this study was at the higher end compared with previous studies 22 23 ; almost one-third of PE patients had a diagnosis of cancer registered within 7 years or on the same date as the PE. This was likely due to the exclusion of younger patients (<50 years of age). Cancer-related mortality has been reported to decrease in the general Swedish population, 24 including among middle-aged and older individuals. 25 The findings of the present study suggest that this trend applies primarily to PE patients with known cancer at baseline. In contrast, an opposing trend was observed in PE patients without known cancer at baseline, who experienced an increase in cancer-related mortality over time. This unexpected finding remains unexplained. To our knowledge, no previously published data indicate an increasing prevalence of occult cancer among patients with VTE. We believe that this finding should be interpreted with caution. Potential explanations include an increased detection of incidental PEs in patients undergoing investigation for suspected cancer, attributable to the higher resolution of computed tomography scans.

Cardiovascular mortality in PE patients declined over the study period, aligning with trends observed in the Swedish general population 13 and the general population across high-income European countries. 12 This was also in line with a decreasing prevalence of cardiovascular comorbidities in PE patients over the study period, as well as a decreasing cardiovascular mortality among controls.

The 30-day mortality from fatal VTE decreased over the study period, with the most pronounced decline occurring before the last time period. However, interpreting the low VTE mortality during the Covid-19 pandemic (2020–2021) is challenging, as many Covid-19 patients were not subjected to diagnostic imaging for PE. In this context, fatal PEs may have been misclassified as Covid-19-related mortality on death certificates. In comparing our PE-related mortality data with previous studies, it is important to note that our cohort includes only patients with a hospital-verified diagnosis, excluding those first identified through cause-of-death records, which differs from some prior datasets. 8 26 A previous U.S. study reported an increase in PE-related mortality during the Covid-19 pandemic; however, PE diagnoses in any position on the cause-of-death certificate were included. 26 In contrast, our analysis was restricted to cases where PE was designated as the underlying cause of death. A possible explanation for our reported declining mortality due to fatal VTE could be therapeutic advancements in high-risk patients, including catheter-directed treatment and extracorporeal membrane oxygenation. However, these therapeutic options were only available at a few Swedish facilities during the study period and are therefore unlikely to have had a major impact. It is also possible that the classification of PE patients in different risk groups has improved the identification of the most critically ill patients. 27 However, the most plausible factor driving the decline in 30-day mortality due to fatal VTE is the widespread use of computed tomography pulmonary angiography (CTPA). Readily available CTPA allows earlier identification and treatment of smaller PEs, preventing the development of large clots as well as fast diagnosis of larger, life-threatening clots. Nevertheless, given the lack of autopsy data or chart reviews of patients, these results should be interpreted with caution.

Bleeding contributed minimally to mortality, ranging from 0.1 to 0.2% in 30-day mortality and 0.2% in 31- to 365-day mortality. In 2018–2022, 0.2% of PE patients died from fatal VTE within the 31- to 365-day mortality period. Hence, under current anticoagulant treatment protocols, patients with PE rarely succumb to either bleeding or VTE.

Our interpretation of high all-cause mortality for PE patients, despite low fatal VTE incidence, is that PE serves as a marker of high mortality risk rather than a direct cause of death. This aligns with a previous meta-analysis of randomized controlled trials assessing primary and secondary prevention of VTE, showing that prevention of VTE does not seem to impact overall mortality. 28

## Strengths and Limitations

Among the strengths of this study is its large, nationwide sample from high-quality registers, covering all socioeconomic groups—in contrast to studies based on health care insurance databases.

Our study also has several limitations related to the use of register data. The PE diagnoses in the present study were not validated by radiology reports. However, a previous validation study has suggested a high positive predictive value of PE diagnoses in the National Patient Register. 29 Another limitation is the imprecision of death certificate data. However, previous data suggest a strong agreement between medical records and the National Cause of Death Register 18 data. Fatal PE is a difficult diagnosis in the absence of an autopsy. However, this is not exclusive to studies utilizing death register data but also applies to cohort studies and randomized trials. 30

## Conclusion

Cancer was the main driver of both 30-day and 31- to 365-day mortality in patients with first-time PE aged ≥50 years, showing no significant change over time. However, while patients with known cancers at baseline had a decreasing cancer-related mortality, patients without cancer at baseline had an unexpected increase in cancer-related mortality. This finding requires further studies.

In the last decade, despite a high all-cause mortality, only a small proportion of deaths were caused by fatal VTE or bleeding, in particular for the 31- to 365-day mortality. This suggests that PE may act as a marker of high mortality rather than a direct cause of mortality. Consequently, further advancements in PE treatment are unlikely to substantially impact overall mortality despite potential improvements in PE and bleeding-related mortality.

## Notes

**What is known about this topic?**

- Pulmonary embolism (PE) is associated with high 30-day all-cause mortality, although this has been decreasing over time. In contrast, 1-year mortality remains high and stable.
- Cancer, PE, and infection have been identified as the primary causes of 30-day mortality, whereas cancer, cardiovascular disease, and recurrent PE are the leading causes of 1-year mortality.
- The impact of recent advancements in cancer, cardiovascular, and PE care on cause-specific mortality following a PE remains unclear.

**What does this paper add?**

- Cancer-related mortality in PE patients has remained stable overall; however, subgroup analysis revealed a decrease among patients with known cancer and an increase among those without.
- In recent years, few PE patients have died due to VTE or bleeding, suggesting that PE is more a marker of high mortality risk than a direct cause. Therefore, further PE treatment advancements may have limited impact on overall mortality.
