# Supplementary material for: Time Trends in Cause-Specific Mortality in Patients with Pulmonary Embolism Aged 50 Years and Older
Source: Thromb Haemost. 2025 Aug 8;126(6):595–609. doi: 10.1055/a-2668-5296 (PMC13288343; doi:10.1055/a-2668-5296)

Supplementary Material

Time trends in cause-specific mortality in patients with pulmonary embolism aged 50 years and older.

Katarina Glise Sandblad[1,2], Kristina Svennerholm[3,4], Jacob Philipson[1,2], Maria Roupe[1,5] Aldina Pivodic[6], Andrea Dahl Sturedahl[6], Carl Johan Svensson[3,4], Sam Schulman[7], Mazdak Tavoly[8,9]

[1]Department of Molecular and Clinical Medicine, Institute of Medicine, Sahlgrenska Academy, University of Gothenburg, Gothenburg, Sweden.
[2]Region Västra Götaland, Sahlgrenska University Hospital/Östra, Department of Medicine, Geriatrics and Emergency Medicine, Gothenburg, Sweden.
[3]Department of Anaesthesiology and Intensive Care, Institute of Clinical Sciences, Sahlgrenska Academy, University of Gothenburg, Gothenburg, Sweden
[4]Region Västra Götaland, Sahlgrenska University Hospital, Department of Anaesthesiology and Intensive Care, Gothenburg, Sweden
[5]Department of Medicine, Region Västra Götaland, Sahlgrenska University Hospital/Mölndal, Gothenburg, Sweden.
[6]APNC Sweden, Gothenburg, Sweden
[7]Department of Medicine and Thrombosis and Atherosclerosis Research Institute, McMaster University, Hamilton, ON, Canada
[8]Region Västra Götaland, Sahlgrenska University Hospital/Sahlgrenska, Department of Medicine, Geriatrics and Emergency Medicine, Gothenburg, Sweden
[9]Department of Research, Østfold Hospital, Sarpsborg, Norway

Corresponding author:

Katarina Glise Sandblad
Department of Molecular and Clinical Medicine
Institute of Medicine
Sahlgrenska University Hospital/Östra
Göteborg SE 416 85
Sweden
E-mail: katarina.glise.sandblad@vgregion.se
Phone: +46707708082

Table of contents

	Page
Supplementary Table 1<br>Codes for comorbidities, temporary provoking factors, anticoagulants, and concomitant medications.	3
Supplementary Table 2<br>Codes for grouped mortality causes.	5
Supplementary Table 3<br>Baseline characteristics for controls.	6
Supplementary Table 4.<br>Relative risk (RR) in mortality per one unit change in calendar year	8
Supplementary Table 5<br>30-day and 31-365-day mortality causes among controls.	10
Supplementary Table 6<br>30-day and 31-365-day mortality causes PE patients with cancer.	13
Supplementary Table 7<br>30-day and 31-365-day mortality causes PE patients without cancer.	16
Supplementary Table 8<br>30-day and 31-365-day mortality causes PE patients with temporary provoking factors	19
Supplementary Figure 1.<br>Mortality causes for 30-day mortality in all patient groups and controls during different time periods.	22
Supplementary Figure 2.<br>Mortality causes for 31-365 day mortality in all patient groups and controls during different time periods.	23

Supplementary Table 1: Comorbidities (within 7 years before or on the same date as the VTE), temporary provoking factors (within 3 months or on the same date as the VTE), anticoagulant treatment defined as dispensed within 30 days from index date and concomitant medications (within 3 months from PE).

Variable	ICD 10/ATC/KVÅ
**Comorbidities**	
Cancer	C00-C97
Ischemic heart disease	I20-I25
Heart failure	I50
Peripheral artery disease	I73.9, I70.2
Ischemic stroke	I63, I64
Hemorrhagic stroke	I60-I62
Dementia	F00-F03
Chronic obstructive pulmonary disease	J40-J44
Systemic connective tissue disorders	M30-M36
Peptic ulcer	M30-M36
Liver disease	K70-K77
Diabetes mellitus	E10-E14
Kidney failure	N17-N19
HIV	B20-B24
Bleeding	I60-62, S06.4-S06.6, I31.2, I85.0, I98.3, K25.0, K25.2, K26.0, K26.2, K27.0, K27.2, K28.0, K28.2, K62.5, K92.0, K92.1, K92.2, R04, D50.0, D62.9, N93, R31
Psychosis	F23
Chronic obstructive pulmonary disease	J40-J44
Systemic connective tissue disorders	M30-M36
Peptic ulcer	K25-K28
**Temporary provoking factors**	
Covid-19	U07.1, U07.2

Surgery	Classification of Healthcare Interventions (KVÅ) A-B, F-H, J-M, P, Y
Lower extremity fracture	S72, S82, S92, T12
Trauma	V01-X59
Hormone replacement therapy	ATC: G03C, G03F
**Anticoagulant treatment**	Anatomical Therapeutic Chemical (ATC) Classification
Warfarin	B01AA03
Apixaban	B01AF02
Rivaroxaban	B01AF01 (excluding Rivaroxaban 2,5 mg)
Edoxaban	B01AF03
Dabigatran	B01AE07
Low molecular weight heparin	B01AB04, B01AB05, B01AB09, B01AB10
**Other concomitant treatment**	
Antiplatelet treatment	B01AC
Proton pump inhibitors	A02BC
Statins	C10AA
Selective serotonin reuptake inhibitor (SSRI)	N06AB
ICD: International classification of diseases, KVÅ: Classification of Healthcare Interventions, ATC: Anatomical Therapeutic Chemical Classification	

Supplementary Table 2: Grouped mortality causes, International Classification of Diseases (ICD)-10 codes.

Neuropsychiatric conditions	F01-F99, G06-G98 (minus G14), U07.0, X41, X42, X44, X45
Cardiovascular diseases	I00-I99 except I26, I80-I82, I312, I60, I61, I62, I850, I983,
Respiratory diseases	J30-J98
Malignant neoplasms	C00-C97
VTE	I26, I80, I81-82
Bleeding	I60-62, S06.4-S06.6, I31.2, I85.0, I98.3, K25.0, K25.2, K26.0, K26.2, K27.0, K27.2, K28.0, K28.2, K62.5, K92.2, K92.0, K92.1, R04, D50.0, D62.9, N93, R31
Injuries	V01-Y89 (minus X41-X42, X44-X45), U12.9
Infectious and parasitic diseases	A00-B99, G00-G04, G14, N70-N73, P37.3, P37.4
Covid-19	U071 ,U072
Other	All other causes of death

Supplementary Table 3. Baseline characteristics for pulmonary embolism (PE) controls during three different time periods: 2006-2011, 2012-2017 and 2018-2023.

	2006-2011	2012-2017	2018-2023
**Number of matched PE controls**	**N=146239**	**N=176108**	**N=199514**
Age, median (range)	74 (50 - 104)	73 (50 - 105)	73 (32 - 109)
**Age category**			
50-64	34333 (23.5%)	40472 (23.0%)	49413 (24.8%)
65-79	62670 (42.9%)	83295 (47.3%)	96867 (48.6%)
≥80	49236 (33.7%)	52341 (29.7%)	53234 (26.7%)
Sex			
Female	77307 (52.9%)	91144 (51.8%)	101912 (51.1%)
**Comorbidities**			
Cancer	18068 (12.4%)	25149 (14.3%)	29261 (14.7%)
Ischemic heart disease	16190 (11.1%)	17332 (9.8%)	13237 (6.6%)
Heart failure	6786 (4.6%)	6336 (3.6%)	3966 (2.0%)
Peripheral arterial disease	2409 (1.6%)	2684 (1.5%)	2330 (1.2%)
Ischemic stroke	5796 (4.0%)	5480 (3.1%)	3079 (1.5%)
Hemorrhagic stroke	1072 (0.7%)	1341 (0.8%)	1058 (0.5%)
Dementia	3744 (2.6%)	4676 (2.7%)	3210 (1.6%)
COPD	3910 (2.7%)	5245 (3.0%)	4402 (2.2%)
Systemic connective tissue disorders	2386 (1.6%)	3277 (1.9%)	2879 (1.4%)
Peptic ulcer	140 (0.1%)	126 (0.1%)	74 (0.0%)
Liver disease	664 (0.5%)	911 (0.5%)	1265 (0.6%)
Diabetes	10913 (7.5%)	14940 (8.5%)	14439 (7.2%)
Renal disease	1814 (1.2%)	3315 (1.9%)	3664 (1.8%)
HIV	26 (0.0%)	73 (0.0%)	144 (0.1%)
Bleeding	666 (0.5%)	772 (0.4%)	790 (0.4%)
Psychosis	137 (0.1%)	196 (0.1%)	137 (0.1%)
**Temporary provoking factors**			
Any temporary provoking factor	13800 (9.4%)	15712 (8.9%)	17355 (8.7%)
Covid			1125 (0.6%)
Surgery	3354 (2.3%)	4002 (2.3%)	4094 (2.1%)
Lower extremity fracture	528 (0.4%)	494 (0.3%)	410 (0.2%)
Trauma	1296 (0.9%)	1701 (1.0%)	1799 (0.9%)

Hormone replacement therapy	9498 (6.5%)	10461 (5.9%)	10945 (5.5%)
**Anticoagulant treatment***			
Warfarin	2406 (1.6%)	4079 (2.3%)	2660 (1.3%)
Apixaban	0 (0.0%)	627 (0.4%)	5171 (2.6%)
Rivaroxaban	40 (0.0%)	713 (0.4%)	2000 (1.0%)
Edoxaban	0 (0.0%)	0 (0.0%)	69 (0.0%)
Dabigatran	77 (0.1%)	779 (0.4%)	1209 (0.6%)
Low molecular weight heparin	5272 (3.6%)	16544 (9.4%)	26778 (13.4%)
**Other concomitant treatment**			
Antiplatelet treatment	47779 (32.7%)	57202 (32.5%)	56704 (28.4%)
Proton pump inhibitor	34848 (23.8%)	64577 (36.7%)	88770 (44.5%)
Statins	37351 (25.5%)	61724 (35.0%)	77819 (39.0%)
Selective serotonin reuptake inhibitors	18793 (12.9%)	30053 (17.1%)	38378 (19.2%)

Data are presented as median (range) and number of observations, or number (percentage).

* Anticoagulant treatment is defined as dispensed within 3 months following index date.
COPD: Chronic obstructive pulmonary disease.

Supplementary Table 4. Relative risk (RR) per one unit change in calendar year among pulmonary embolism (PE) patients, controls, and subgroups of PE patients.

		All PE patients	Controls	PE patients with cancer	PE patients without cancer	PE patients with temporary provoking factors
**Outcome**	**Time period outcome**	**RR***	**RR***	**RR***	**RR***	**RR***
**Death by cancer**	**0-30 days**	1.00 (0.99 - 1.01)	1.00 (0.97 - 1.02)	0.98 (0.98 - 0.99)	1.12 (1.10 - 1.13)	1.00 (0.99 - 1.01)
		p=0.96	p=0.82	p=<.0001	p=<.0001	p=0.78
	**31-365 days**	1.00 (0.99 - 1.00)	1.01 (1.00 - 1.01)	0.98 (0.98 - 0.99)	1.06 (1.05 - 1.07)	0.99 (0.99 - 1.00)
		p=0.037	p=0.11	p=<.0001	p=<.0001	p=0.11
**Cardiovascular death**	**0-30 days**	0.94 (0.93 - 0.94)	0.95 (0.94 - 0.97)	0.94 (0.92 - 0.96)	0.94 (0.93 - 0.94)	0.95 (0.93 - 0.96)
		p=<.0001	p=<.0001	p=<.0001	p=<.0001	p=<.0001
	**31-365 days**	0.96 (0.95 - 0.96)	0.96 (0.96 - 0.97)	0.96 (0.94 - 0.97)	0.96 (0.95 - 0.96)	0.96 (0.95 - 0.98)
		p=<.0001	p=<.0001	p=<.0001	p=<.0001	p=<.0001
**Death by respiratory diseases**	**0-30 days**	1.04 (1.02 - 1.05)	1.00 (0.95 - 1.05)	1.05 (1.01 - 1.09)	1.03 (1.02 - 1.05)	1.05 (1.01 - 1.08)
		p=<.0001	p=0.99	p=0.0084	p=0.0002	p=0.0033
	**31-365 days**	1.01 (0.99 - 1.02)	1.03 (1.01 - 1.05)	1.03 (1.00 - 1.05)	1.00 (0.99 - 1.02)	1.01 (0.99 - 1.04)
		p=0.34	p=0.0005	p=0.07	p=0.83	p=0.27
**Neuropsychiatric death**	**0-30 days**	1.02 (1.00 - 1.04)	1.04 (1.01 - 1.07)	1.04 (0.99 - 1.10)	1.02 (1.00 - 1.04)	1.01 (0.98 - 1.04)
		p=0.017	p=0.0025	p=0.09	p=0.06	p=0.61
	**31-365 days**	1.05 (1.04 - 1.07)	1.03 (1.02 - 1.04)	1.05 (1.01 - 1.09)	1.05 (1.04 - 1.07)	1.04 (1.02 - 1.07)
		p=<.0001	p=<.0001	p=0.012	p=<.0001	p=0.0012
**Death by fatal VTE**	**0-30 days**	0.94 (0.93 - 0.95)	0.94 (0.80 - 1.10)	0.94 (0.92 - 0.96)	0.94 (0.93 - 0.95)	0.95 (0.93 - 0.97)
		p=<.0001	p=0.45	p=<.0001	p=<.0001	p=<.0001
	**31-365 days**	0.95 (0.93 - 0.96)	1.00 (0.91 - 1.08)	0.94 (0.91 - 0.98)	0.95 (0.93 - 0.97)	0.93 (0.90 - 0.96)
		p=<.0001	p=0.92	p=0.0039	p=<.0001	p=<.0001
**Death by bleeding**	**0-30 days**	0.99 (0.97 - 1.02)	0.93 (0.85 - 1.01)	0.98 (0.91 - 1.04)	1.00 (0.97 - 1.03)	1.02 (0.98 - 1.07)
		p=0.67	p=0.08	p=0.47	p=0.89	p=0.39
	**31-365 days**	1.00 (0.97 - 1.02)	1.00 (0.97 - 1.02)	0.98 (0.93 - 1.03)	1.00 (0.97 - 1.04)	1.00 (0.95 - 1.05)
		p=0.78	p=0.95	p=0.50	p=0.91	p=0.97
**Death by covid-19**	**0-30 days**	1.47 (1.40 - 1.53)	1.51 (1.22 - 1.86)	1.54 (1.39 - 1.71)	1.45 (1.38 - 1.52)	1.44 (1.36 - 1.52)
		p=<.0001	p=0.0001	p=<.0001	p=<.0001	p=<.0001
	**31-365 days**	1.63 (1.51 - 1.75)	1.54 (1.44 - 1.65)	1.60 (1.37 - 1.88)	1.64 (1.50 - 1.78)	1.56 (1.41 - 1.73)
		p=<.0001	p=<.0001	p=<.0001	p=<.0001	p=<.0001
**Death by injury**	**0-30 days**	0.94 (0.92 - 0.95)	0.97 (0.91 - 1.02)	0.94 (0.91 - 0.97)	0.94 (0.92 - 0.96)	0.93 (0.91 - 0.94)
		p=<.0001	p=0.23	p=<.0001	p=<.0001	p=<.0001
	**31-365 days**	0.99 (0.97 - 1.01)	1.01 (0.99 - 1.03)	0.99 (0.94 - 1.04)	0.99 (0.96 - 1.01)	1.00 (0.97 - 1.03)
		p=0.32	p=0.16	p=0.75	p=0.36	p=1.00

**All-cause mortality**	**0-30 days**	0.98 (0.98 - 0.99)	0.99 (0.98 - 1.00)	0.98 (0.98 - 0.99)	0.99 (0.98 - 0.99)	0.99 (0.98 - 0.99)
		p=<.0001	p=0.10	p=<.0001	p=<.0001	p=<.0001
	**31-365 days**	0.99 (0.99 - 0.99)	1.00 (0.99 - 1.00)	0.98 (0.98 - 0.98)	1.01 (1.00 - 1.01)	1.00 (0.99 - 1.00)
		p=<.0001	p=0.029	p=<.0001	p=0.0019	p=0.07
Poisson regression was used to calculate RR						
* RR per one unit change in calendar year, adjusted for age and sex.						

Supplementary Table 5. 30-day and 31-365-day mortality causes and all-cause mortality among controls during different time periods. Data presented with number of events, percentage, and event rate per 1000 person-years.

Mortality cause		2006-2011	2012-2017	2018-2023
Cancer	**30-day mortality**			
	n/N (%)	79/146239 (0.1%)	90/176108 (0.1%)	95/199396 (0.0%)
	Event rate (95% CI) per 1000 person-years	6.4 (5.0-7.9)	6.0 (4.8-7.4)	5.6 (4.5-6.9)
	**31-365-day mortality**			
	n/N (%)	804/145803 (0.6%)	1013/175599 (0.6%)	928/170994 (0.5%)
	Event rate (95% CI)***	6.1 (5.7-6.5)	6.4 (6.0-6.8)	6.0 (5.6-6.4)
Cardiovascular diseases	**30-day mortality**			
	n/N (%)	197/146239 (0.1%)	190/176108 (0.1%)	131/199396 (0.1%)
	Event rate (95% CI)***	15.9 (13.8-18.3)	12.7 (11.0-14.7)	8.1 (7.6-8.6)
	**31-365-day mortality**			
	n/N (%)	1947/145803 (1.3%)	2016/175599 (1.1%)	1252/170994 (0.7%)
	Event rate (95% CI)***	14.8 (14.1-15.5)	12.7 (12.2-13.3)	8.1 (7.6-8.6)
Respiratory diseases	**30-day mortality**			
	n/N (%)	15/146239 (0.0%)	23/176108 (0.0%)	21/199396 (0.0%)
	Event rate (95% CI)***	1.2 (0.7-2.0	1.5 (1.0-2.3)	1.2 (0.8-1.9)
	**31-365-day mortality**			
	n/N (%)	170/145803 (0.1%)	238/175599 (0.1%)	241/170994 (0.1%)
	Event rate (95% CI)***	1.3 (1.1-1.5)	1.5 (1.3-1.7)	1.6 (1.4-1.8)
Neuropsychiatric conditions**	**30-day mortality**			
	n/N (%)	48/146239 (0.0%)	80/176108 (0.0%)	94/199396 (0.0%)
	Event rate (95% CI)***	3.9 (2.9-5.1)	5.4 (4.2-6.7)	5.6 (4.5-6.8)
	**31-365-day mortality**			
	n/N (%)	546/145803 (0.4%)	889/175599 (0.5%)	793/170994 (0.5%)

	**Event rate (95% CI)***	4.1 (3.8-4.5)	5.6 (5.2-6.0)	5.1 (4.8-5.5)
**Fatal venous thromboembolism**	**30-day mortality**			
	**n/N (%)**	3/146239 (0.0%)	1/176108 (0.0%)	2/199396 (0.0%)
	**Event rate (95% CI)***	0.2 (0.0-0.7)	0.1 (0.0-0.4)	0.1 (0.0-0.4)
	**31-365-day mortality**			
	**n/N (%)**	8/145803 (0.0%)	6/175599 (0.0%)	8/170994 (0.0%)
	**Event rate (95% CI)***	0.1 (0.0-0.1)	0.0 (0.0-0.1)	0.1 (0.0-0.1)
**Bleeding**	**30-day mortality**			
	**n/N (%)**	9/146239 (0.0%)	9/176108 (0.0%)	5/199396 (0.0%)
	**Event rate (95% CI)***	0.7 (0.3-1.4)	0.6 (0.3-1.1)	0.3 (0.1-0.7)
	**31-365-day mortality**			
	**n/N (%)**	81/145803 (0.1%)	90/175599 (0.1%)	80/170994 (0.0%)
	**Event rate (95% CI)***	0.6 (0.5-0.8)	0.6 (0.5-0.7)	0.5 (0.4-0.6)
**Injury**	**30-day mortality**			
	**n/N (%)**	17/146239 (0.0%)	17/176108 (0.0%)	14/199396 (0.0%)
	**Event rate (95% CI)***	1.4 (0.8-2.2)	1.1 (0.7-1.8)	0.8 (0.5-1.4)
	**31-365-day mortality**			
	**n/N (%)**	142/145803 (0.1%)	171/175599 (0.1%)	163/170994 (0.1%)
	**Event rate (95% CI)***	1.1 (0.9-1.3)	1.1 (0.9-1.3)	1.1 (0.9-1.2)
**Infectious and parasitic diseases**	**30-day mortality**			
	**n/N (%)**	5/146239 (0.0%)	11/176108 (0.0%)	11/199396 (0.0%)
	**Event rate (95% CI)***	0.4 (0.1-0.9)	0.7 (0.4-1.3)	0.7 (0.3-1.2)
	**31-365-day mortality**			
	**n/N (%)**	99/145803 (0.1%)	147/175599 (0.1%)	120/170994 (0.1%)
	**Event rate (95% CI)***	0.8 (0.6-0.9)	0.9 (0.8-1.1)	0.8 (0.6-0.9)
**Covid-19**	**30-day mortality**			
	**n/N (%)**	0/146239 (0.0%)	0/176108 (0.0%)	16/199396 (0.0%)

	Event rate (95% CI)***	0	0	0.9 (0.5-1.5)
	31-365-day mortality			
	n/N (%)	0/145803	0/175599	176/170994
		(0.0%)	(0.0%)	(0.1%)
	Event rate (95% CI)***	0	0	1.1 (1.0-1.3)
Other causes	30-day mortality			
	n/N (%)	63/146239	87/176108	89/199396
		(0.0%)	(0.0%)	(0.0%)
	Event rate (95% CI)***	5.1 (3.9-6.5)	5.8 (4.7-7.2)	5.3 (4.2-6.5)
	31-365-day mortality			
	n/N (%)	778/145803	814/175599	709/170994
		(0.5%)	(0.5%)	(0.4%)
	Event rate (95% CI)***	5.9 (5.5-6.3)	5.1 (4.8-5.5)	4.6 (4.3-4.9)
All-cause mortality	30-day mortality			
	n/N (%)	436/146239	508/176108	478/199396
		(0.3%)	(0.3%)	(0.2%)
	Event rate (95% CI)***	35.2 (32.0-38.6	34.0 (31.1-37.1)	28.3 (25.8-30.9)
	31-365-day mortality			
	n/N (%)	4575/145803	5384/175599	4470/170994
		(3.1%)	(3.1%)	(2.6%)
	Event rate (95% CI)***	34.7 (33.7-35.8)	33.9 (33.0-34.9)	28.9 (28.0-29.7)

Confidence interval for unadjusted event rates per 1000 person years are obtained from exact Poisson confidence limits. *For 31-365-day mortality, only numbers for 2008-2022, not 2023. **Dementia, psychiatric illness, neurological illness. *** per 1000 person-years.

Supplementary Table 6. 30-day and 31-365-day mortality causes and all-cause mortality among pulmonary embolism (PE) patients with cancer during different time periods. Data presented with number of events, percentage, and event rate per 1000 person-years.

Mortality cause		2006-2011	2012-2017	2018-2023
Cancer	**30-day mortality**			
	n/N (%)	1351/10013 (13.5%)	1576/12853 (12.3%)	1561/13934 (11.2%)
	Event rate (95% CI) per 1000 person-years	1773.7 (1680.3-1870.8)	1586.2 (1508.9-1666.5)	1434.9 (1364.6-1507.9)
	**31-365-day mortality**			
	n/N (%)	2782/8168 (34.1%)	3415/10745 (31.8%)	2896/10143 (28.6%)
	Event rate (95% CI)***	514.8 (495.9-534.3)	468.5 (452.9-484.4)	407.6 (392.9-422.7)
Cardiovascular diseases	**30-day mortality**			
	n/N (%)	144/10013 (1.4%)	149/12853 (1.2%)	107/13934 (0.8%)
	Event rate (95% CI)***	189.0 (159.4-222.6)	150.0 (126.9-176.1)	98.4 (80.6-118.9)
	**31-365-day mortality**			
	n/N (%)	237/8168 (2.9%)	290/10745 (2.7%)	107/13934 (0.8%)
	Event rate (95% CI)***	43.9 (38.5-49.8)	39.8 (35.3-44.6)	98.4 (80.6-118.9)
Respiratory diseases	**30-day mortality**			
	n/N (%)	23/10013 (0.2%)	38/12853 (0.3%)	59/13934 (0.4%)
	Event rate (95% CI)***	30.2 (19.1-45.3)	38.2 (27.1-52.5)	54.2 (41.3-70.0)
	**31-365-day mortality**			
	n/N (%)	54/8168 (0.7%)	90/10745 (0.8%)	93/10143 (0.9%)
	Event rate (95% CI)***	10.0 (7.5-13.0)	12.3 (9.9-15.2)	13.1 (10.6-16.0)
Neuropsychiatric conditions**	**30-day mortality**			
	n/N (%)	10/10013 (0.1%)	21/8168 (0.3%)	33/13934 (0.2%)
	Event rate (95% CI)***	13.1 (6.3-24.1)	24.2 (15.5-35.9)	30.3 (20.9-42.6)
	**31-365-day mortality**			
	n/N (%)	21/8168	54/10745	58/10143

		(0.3%)	(0.5%)	(0.6%)
	**Event rate (95% CI)*** **	3.9 (2.4-5.9)	7.4 (5.6-9.7)	8.2 (6.2-10.6)
**Fatal venous thromboembolism**	**30-day mortality**			
	**n/N (%)**	113/10013 (1.1%)	119/12853 (0.9%)	83/13934 (0.6%)
	**Event rate (95% CI)*** **	148.4 (122.3-178.4)	119.8 (99.2-143.3)	76.3 (60.8-94.6)
	**31-365-day mortality**			
	**n/N (%)**	40/8168 (0.5%)	42/10745 (0.4%)	30/10143 (0.3%)
	**Event rate (95% CI)*** **	7.4 (5.3-10.1)	5.8 (4.2-7.8)	4.2 (2.8-6.0)
**Bleeding**	**30-day mortality**			
	**n/N (%)**	9/10013 (0.1%)	12/12853 (0.1%)	11/13934 (0.1%)
	**Event rate (95% CI)*** **	11.8 (5.4-22.4)	12.1 (6.2-21.1)	10.1 (5.0-18.1)
	**31-365-day mortality**			
	**n/N (%)**	17/8168 (0.2%)	24/10745 (0.2%)	24/10143 (0.2%)
	**Event rate (95% CI)*** **	3.1 (1.8-5.0)	3.3 (2.1-4.9)	3.4 (2.2-5.0)
**Injury**	**30-day mortality**			
	**n/N (%)**	58/10013 (0.6%)	46/12853 (0.4%)	43/13934 (0.3%)
	**Event rate (95% CI)*** **	76.1 (57.8-98.4)	46.3 (33.9-61.8)	39.5 (28.6-53.2)
	**31-365-day mortality**			
	**n/N (%)**	23/8168 (0.3%)	15/10745 (0.1%)	29/10143 (0.3%)
	**Event rate (95% CI)*** **	4.3 (2.7-6.4)	2.1 (1.2-3.4)	4.1 (2.7-5.9)
**Infectious and parasitic diseases**	**30-day mortality**			
	**n/N (%)**	19/10013 (0.2%)	31/12853 (0.2%)	20/13934 (0.1%)
	**Event rate (95% CI)*** **	24.9 (15.0-39.0)	31.2 (21.2-44.3)	18.4 (11.2-28.4)
	**31-365-day mortality**			
	**n/N (%)**	29/8168 (0.4%)	20/10745 (0.2%)	19/10143 (0.2%)

	**Event rate (95% CI)*****	5.4 (3.6-7.7)	2.7 (1.7-4.2)	2.7 (1.6-4.2)
**Covid-19**	**30-day mortality**			
	**n/N (%)**	0/10013 (0.0%)	0/12853 (0.0%)	81/13934 (0.6%)
	**Event rate (95% CI)*****	0	0	74.5 (59.1-92.5)
	**31-365-day mortality**			
	**n/N (%)**	0/8168 (0.0%)	0/10745 (0.0%)	42/10143 (0.4%)
	**Event rate (95% CI)*****	0	0	5.9 (4.3-8.0)
**Other causes**	**30-day mortality**			
	**n/N (%)**	118/10013 (1.2%)	113/12853 (0.9%)	131/13934 (0.9%)
	**Event rate (95% CI)*****	154.9 (128.2-185.5)	113.7 (93.7-136.7)	120.4 (100.7-142.9)
	**31-365-day mortality**			
	**n/N (%)**	147/8168 (1.8%)	180/10745 (1.7%)	150/10143 (1.5%)
	**Event rate (95% CI)*****	27.2 (23.0-32.0)	24.7 (21.2-28.6)	21.1 (17.9-24.8)
**All-cause mortality**	**30-day mortality**			
	**n/N (%)**	1845/10013 (18.4%)	2108/12853 (16.4%)	2129/13934 (15.3%)
	**Event rate (95% CI)*****	2422.2 (2312.9-2535.3)	2121.7 (2032.1-2214.2)	1957.1 (1874.8-2042.0)
	**31-365-day mortality**			
	**n/N (%)**	3350/8168 (41.0%)	4130/10745 (38.4%)	3538/10143 (34.9%)
	**Event rate (95% CI)*****	619.9 (599.1-641.3)	566.5 (549.4-584.1)	497.9 (481.6-514.6)

Confidence interval for unadjusted event rates per 1000 person years are obtained from exact Poisson confidence limits. *For 31-365-day mortality, only numbers for 2008-2022, not 2023. **Dementia, psychiatric illness, neurological illness. *** per 1000 person-years.

Supplementary Table 7. 30-day and 31-365-day mortality causes and all-cause mortality among pulmonary embolism (PE) patients without cancer during different time periods. Data presented with number of events, percentage, and event rate per 1000 person-years.

Mortality cause		2006-2011	2012-2017	2018-2023
Cancer	**30-day mortality**			
	n/N (%)	101/21037 (0.5%)	286/25891 (1.1%)	586/31718 (1.8%)
	Event rate (95% CI) per 1000 person-years	61.0 (49.7-74.2)	137.6 (122.1-154.5)	230.0 (211.7-249.4)
	**31-365-day mortality**			
	n/N (%)	406/18750 (2.2%)	866/23655 (3.7%)	1085/24691 (4.4%)
	Event rate (95% CI)***	25.7 (23.2-28.3)	43.6 (40.8-46.6)	52.5 (49.4-55.7)
Cardiovascular diseases	**30-day mortality**			
	n/N (%)	578/21037 (2.7%)	442/25891 (1.7%)	383/31718 (1.2%)
	Event rate (95% CI)***	349.3 (321.4-379.0)	212.6 (193.2-233.4)	150.3 (135.6-166.1)
	**31-365-day mortality**			
	n/N (%)	877/18750 (4.7%)	809/23655 (3.4%)	616/24691 (2.5%)
	Event rate (95% CI)***	55.4 (51.8-59.2)	40.8 (38.0-43.7)	29.8 (27.5-32.2)
Respiratory diseases	**30-day mortality**			
	n/N (%)	116/21037 (0.6%)	175/25891 (0.7%)	248/31718 (0.8%)
	Event rate (95% CI)***	70.1 (57.9-84.1)	84.2 (72.2-97.6)	97.3 (85.6-110.2)
	**31-365-day mortality**			
	n/N (%)	246/18750 (1.3%)	335/23655 (1.4%)	300/24691 (1.2%)
	Event rate (95% CI)***	15.6 (13.7-17.6)	16.9 (15.1-18.8)	14.5 (12.9-16.2)
Neuropsychiatric conditions**	**30-day mortality**			
	n/N (%)	103/21037 (0.5%)	125/25891 (0.5%)	181/31718 (0.6%)
	Event rate (95% CI)***	62.2 (50.8-75.5)	60.1 (50.0-71.6)	71.0 (61.1-82.2)
	**31-365-day mortality**			
	n/N (%)	137/18750	253/23655	315/24691

		(0.7%)	(1.1%)	(1.3%)
	**Event rate (95% CI)*** **	8.7 (7.3-10.2)	12.7 (11.2-14.4)	15.2 (13.6-17.0)
**Fatal venous thromboembolism**	**30-day mortality**			
	**n/N (%)**	712/21037 (3.4%)	604/25891 (2.3%)	517/31718 (1.6%)
	**Event rate (95% CI)*** **	430.3 (399.3-463.1)	290.5 (267.8-314.6)	202.9 (185.8-221.1)
	**31-365-day mortality**			
	**n/N (%)**	179/18750 (1.0%)	185/23655 (0.8%)	127/24691 (0.5%)
	**Event rate (95% CI)*** **	11.3 (9.7-13.1)	9.3 (8.0-10.8)	6.1 (5.1-7.3)
**Bleeding**	**30-day mortality**			
	**n/N (%)**	41/21037 (0.2%)	41/25891 (0.2%)	61/31718 (0.2%)
	**Event rate (95% CI)*** **	24.8 (17.8-33.6)	19.7 (14.2-26.8)	23.9 (18.3-30.7)
	**31-365-day mortality**			
	**n/N (%)**	39/18750 (0.2%)	49/23655 (0.2%)	55/24691 (0.2%)
	**Event rate (95% CI)*** **	2.5 (1.8-3.4)	2.5 (1.8-3.3)	2.7 (2.0-3.5)
**Injury**	**30-day mortality**			
	**n/N (%)**	218/21037 (1.0%)	161/25891 (0.6%)	150/31718 (0.5%)
	**Event rate (95% CI)*** **	131.8 (114.8-150.5)	77.4 (65.9-90.4)	58.9 (49.8-69.1)
	**31-365-day mortality**			
	**n/N (%)**	73/18750 (0.4%)	86/23655 (0.4%)	82/24691 (0.3%)
	**Event rate (95% CI)*** **	4.6 (3.6-5.8)	4.3 (3.5-5.4)	4.0 (3.2-4.9)
**Infectious and parasitic diseases**	**30-day mortality**			
	**n/N (%)**	65/21037 (0.3%)	75/25891 (0.3%)	75/31718 (0.2%)
	**Event rate (95% CI)*** **	39.3 (30.3-50.1)	36.1 (28.4-45.2)	29.4 (23.1-36.9)
	**31-365-day mortality**			
	**n/N (%)**	73/18750 (0.4%)	98/23655 (0.4%)	83/24691 (0.3%)

	**Event rate (95% CI)***	4.6 (3.6-5.8)	4.9 (4.0-6.0)	4.0 (3.2-5.0)
**Covid-19**	**30-day mortality**			
	**n/N (%)**	0/21037 (0.0%)	0/25891 (0.0%)	311/31718 (1.0%)
	**Event rate (95% CI)***	0	0	122.0 (108.9-136.4)
	**31-365-day mortality**			
	**n/N (%)**	0/18750 (0.0%)	0/23655 (0.0%)	158/24691 (0.6%)
	**Event rate (95% CI)***	0	0	7.6 (6.5-8.9)
**Other causes**	**30-day mortality**			
	**n/N (%)**	353/21037 (1.7%)	327/25891 (1.3%)	393/31718 (1.2%)
	**Event rate (95% CI)***	213.3 (191.7-236.8)	157.3 (140.7-175.3)	154.2 (139.3-170.2)
	**31-365-day mortality**			
	**n/N (%)**	362/18750 (1.9%)	447/23655 (1.9%)	390/24691 (1.6%)
	**Event rate (95% CI)***	22.9 (20.6-25.4)	22.5 (20.5-24.7)	18.9 (17.0-20.8)
**All-cause mortality**	**30-day mortality**			
	**n/N (%)**	2287/21037 (10.9%)	2236/25891 (8.6%)	2905/31718 (9.2%)
	**Event rate (95% CI)***	151.2 (145.2-157.4)	1075.4 (1031.3-1121.0)	1140.0 (1098.9-1182.2)
	**31-365-day mortality**			
	**n/N (%)**	2392/18750 (12.8%)	3128/23655 (13.2%)	3211/24691 (13.0%)
	**Event rate (95% CI)***	34.7 (33.7-35.8)	157.6 (152.2-163.3)	155.3 (150.0-160.8)

Confidence interval for unadjusted event rates per 1000 person years are obtained from exact Poisson confidence limits. *For 31-365-day mortality, only numbers for 2008-2022, not 2023. **Dementia, psychiatric illness, neurological illness. *** per 1000 person-years.

Supplementary Table 8. 30-day and 31-365-day mortality causes and all-cause mortality among pulmonary embolism (PE) patients with temporary provoking factors during different time periods. Data presented with number of events, percentage, and event rate per 1000 person-years.

Mortality cause		2006-2011	2012-2017	2018-2023
Cancer	**30-day mortality**			
	n/N (%)	468/9181 (5.1%)	690/11181 (6.2%)	776/14607 (5.3%)
	Event rate (95% CI) per 1000 person-years	660.2 (601.7-722.8)	787.8 (730.1-848.8)	674.3 (627.7-723.5)
	**31-365-day mortality**			
	n/N (%)	1062/7837 (13.6%)	1447/9662 (15.0%)	1411/10864 (13.0%)
	Event rate (95% CI)***	174.6 (164.2-185.4)	195.6 (185.6-205.9)	166.7 (158.1-175.6)
Cardiovascular diseases	**30-day mortality**			
	n/N (%)	195/9181 (2.1%)	206/11181 (1.8%)	165/14607 (1.1%)
	Event rate (95% CI)***	275.1 (237.8-316.5)	235.2 (204.2-269.6)	143.4 (122.3-167.0)
	**31-365-day mortality**			
	n/N (%)	300/7837 (3.8%)	298/9662 (3.1%)	267/10864 (2.5%)
	Event rate (95% CI)***	49.3 (43.9-55.2)	40.3 (35.8-45.1)	31.5 (27.9-35.6)
Respiratory diseases	**30-day mortality**			
	n/N (%)	31/9181 (0.3%)	60/11181 (0.5%)	91/14607 (0.6%)
	Event rate (95% CI)***	43.7 (29.7-62.1)	68.5 (52.3-88.2)	79.1 (63.7-97.1)
	**31-365-day mortality**			
	n/N (%)	66/7837 (0.8%)	94/9662 (1.0%)	103/10864 (0.9%)
	Event rate (95% CI)***	10.8 (8.4-13.8)	12.7 (10.3-15.5)	12.2 (9.9-14.8)
Neuropsychiatric conditions**	**30-day mortality**			
	n/N (%)	31/9181 (0.3%)	40/11181 (0.4%)	58/14607 (0.4%)
	Event rate (95% CI)***	43.7 (29.7-62.1)	45.7 (32.6-62.2)	50.4 (38.3-65.2)

	31-365-day mortality			
	n/N (%)	47/7837 (0.6%)	105/9662 (1.1%)	115/10864 (1.1%)
	Event rate (95% CI)***	7.7 (5.7-10.3)	14.2 (11.6-17.2)	13.6 (11.2-16.3)
Fatal venous thromboembolism	30-day mortality			
	n/N (%)	164/9181 (1.8%)	148/11181 (1.3%)	137/14607 (0.9%)
	Event rate (95% CI)***	231.3 (197.3-269.6)	169.0 (142.8-198.5)	119.0 (99.9-140.7)
	31-365-day mortality			
	n/N (%)	66/7837 (0.8%)	56/9662 (0.6%)	37/10864 (0.3%)
	Event rate (95% CI)***	10.8 (8.4-13.8)	7.6 (5.7-9.8)	4.4 (3.1-6.0)
Bleeding	30-day mortality			
	n/N (%)	18/9181 (0.2%)	24/11181 (0.2%)	36/14607 (0.2%)
	Event rate (95% CI)***	25.4 (15.0-40.1)	27.4 (17.6-40.8)	31.3 (21.9-43.3)
	31-365-day mortality			
	n/N (%)	19/7837 (0.2%)	26/9662 (0.3%)	28/10864 (0.3%)
	Event rate (95% CI)***	3.1 (1.9-4.9)	3.5 (2.3-5.1)	3.3 (2.2-4.8)
Injury	30-day mortality			
	n/N (%)	235/9181 (2.6%)	142/11181 (1.3%)	159/14607 (1.1%)
	Event rate (95% CI)***	331.5 (290.5-376.7)	162.1 (136.6-191.1)	138.2 (117.5-161.4)
	31-365-day mortality			
	n/N (%)	44/7837 (0.6%)	48/9662 (0.5%)	59/10864 (0.5%)
	Event rate (95% CI)***	7.2 (5.3-9.7)	6.5 (4.8-8.6)	7.0 (5.3-9.0)
Infectious and parasitic diseases	30-day mortality			
	n/N (%)	19/9181 (0.2%)	36/11181 (0.3%)	30/14607 (0.2%)
	Event rate (95% CI)***	26.8 (16.1-41.9)	41.1 (28.8-56.9)	26.1 (17.6-37.2)
	31-365-day mortality			
	n/N (%)	26/7837 (0.3%)	41/9662 (0.4%)	30/10864 (0.3%)
	Event rate (95% CI)***	4.3 (2.8-6.3)	5.5 (4.0-7.5)	3.5 (2.4-5.1)

**Covid-19**	**30-day mortality**			
	**n/N (%)**	0/9181 (0.0%)	0/11181 (0.0%)	256/14607 (1.8%)
	**Event rate (95% CI)*** **	0	0	222.4 (196.0-251.4)
	**31-365-day mortality**			
	**n/N (%)**	0/7837 (0.0%)	0/9662 (0.0%)	106/10864 (1.0%)
	**Event rate (95% CI)*** **	0	0	12.5 (10.3-15.1)
**Other causes**	**30-day mortality**			
	**n/N (%)**	183/9181 (2.0%)	173/11181 (1.5%)	198/14607 (1.4%)
	**Event rate (95% CI)*** **	258.2 (222.1-298.4)	197.5 (169.2-229.2)	172.0 (148.9-197.8)
	**31-365-day mortality**			
	**n/N (%)**	158/7837 (2.0%)	227/9662 (2.3%)	207/10864 (1.9%)
	**Event rate (95% CI)*** **	26.0 (22.1-30.4)	30.7 (26.8-34.9)	24.5 (21.2-28.0)
**All-cause mortality**	**30-day mortality**			
	**n/N (%)**	1344/9181 (14.6%)	1519/11181 (13.6%)	1906/14607 (13.0%)
	**Event rate (95% CI)*** **	1895.9 (1795.9-2000.1)	1734.3 (1648.1-1823.7)	1656.2 (1582.7-1732.3)
	**31-365-day mortality**			
	**n/N (%)**	1788/7837 (22.8%)	2342/9662 (24.2%)	2363/10864 (21.8%)
	**Event rate (95% CI)*** **	293.9 (280.5-307.9)	316.5 (303.8-329.6)	279.2 (268.1-290.7)

Confidence interval for unadjusted event rates per 1000 person years are obtained from exact Poisson confidence limits. *For 31-365-day mortality, only numbers for 2008-2022, not 2023. **Dementia, psychiatric illness, neurological illness. *** per 1000 person-years.

Supplementary Figure 1. Mortality causes during different time periods for 30-day mortality for A. all PE patients, B. Controls, C. PE patients with cancer, D. PE patients without cancer, and E. PE patients with temporary provoking factors.

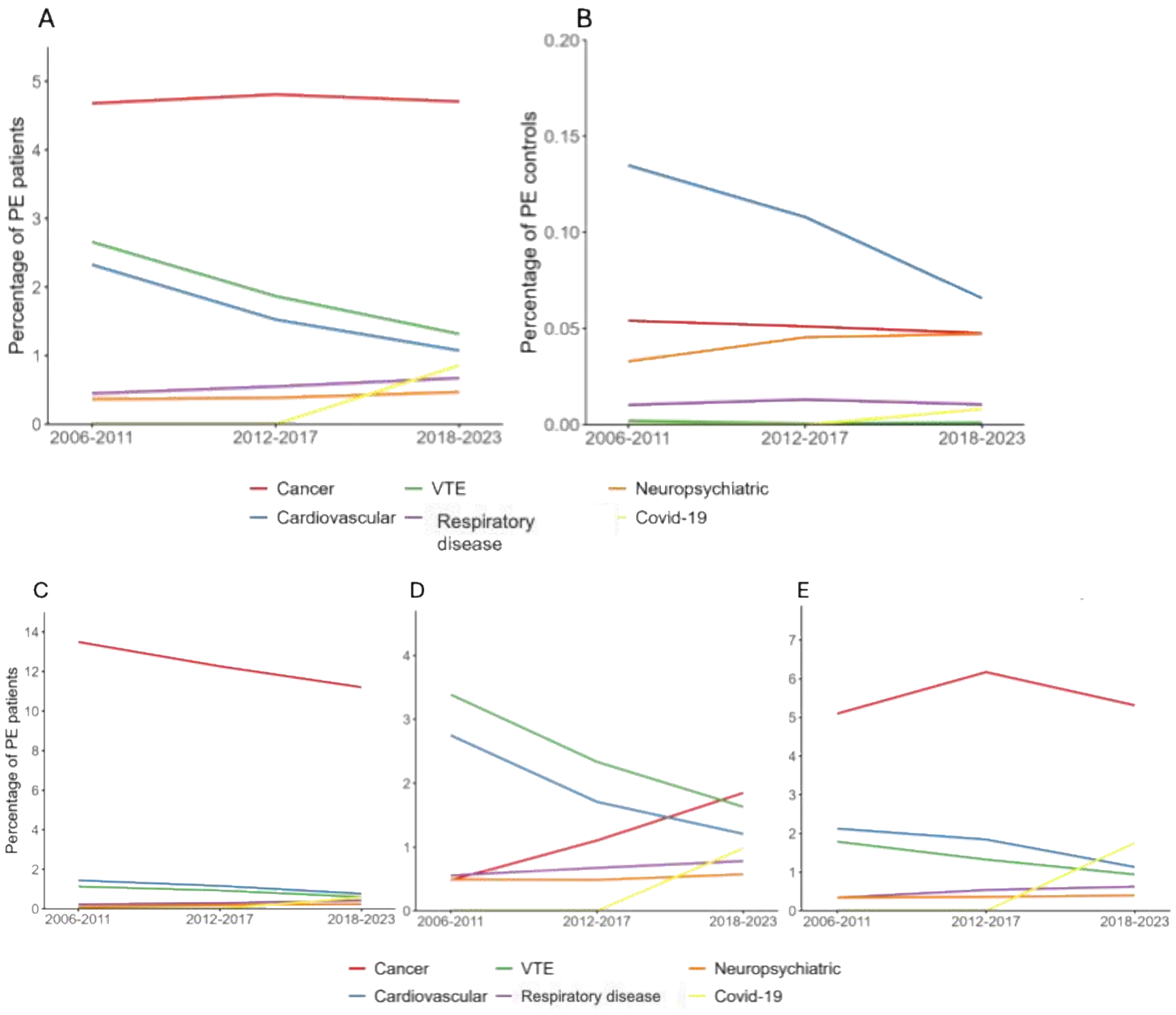

Supplementary Figure 2. Mortality causes during different time periods for 31 to 365-day mortality for A. all PE patients, B. Controls, C. PE patients with cancer, D. PE patients without cancer, and E. PE patients with temporary provoking factors.

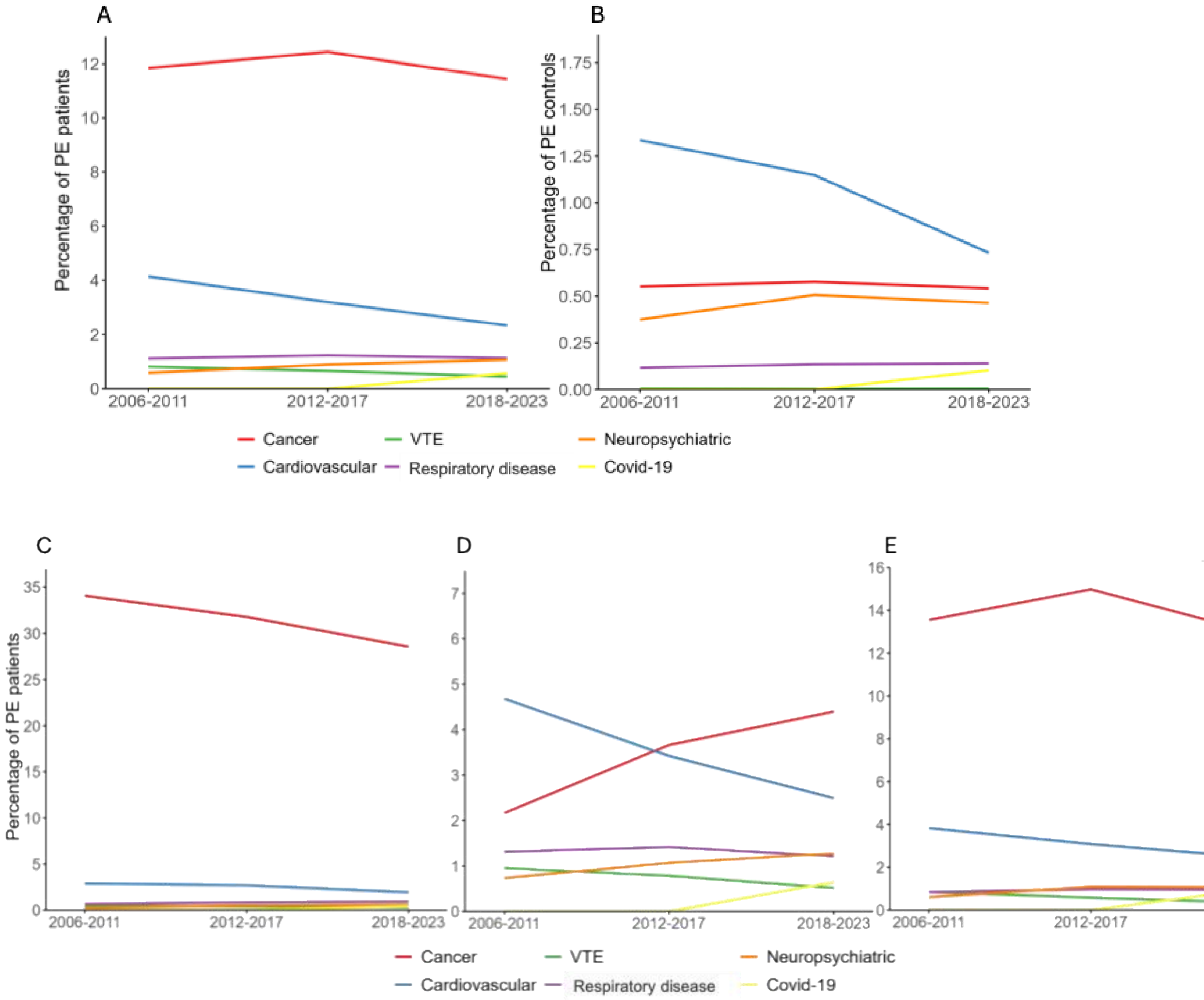

Supplement: Supplementary file 1 — Supplementary Material [file 10-1055-a-2668-5296-s25030145.pdf]
